# Supplementary material for: Pyruvate Dehydrogenase Kinase 1 inhibition mediated oxidative phosphorylation enhancement in cartilage promotes osteoarthritis progression
Source: BMC Musculoskelet Disord. 2023 Jul 20;24:597. doi: 10.1186/s12891-023-06585-6 (PMC10357736; doi:10.1186/s12891-023-06585-6)
Supplement: Supplementary file 1 — Additional file 1. [file 12891_2023_6585_MOESM1_ESM.zip › MF.Gsea.1653627316242/GOMF_OXIDOREDUCTASE_ACTIVITY_ACTING_ON_THE_CH_CH_GROUP_OF_DONORS_NAD_OR_NADP_AS_ACCEPTOR.html]

Details for gene set GOMF\_OXIDOREDUCTASE\_ACTIVITY\_ACTING\_ON\_THE\_CH\_CH\_GROUP\_OF\_DONORS\_NAD\_OR\_NADP\_AS\_ACCEPTOR[GSEA]

|  || Dataset | 55457\_collapsed\_to\_symbols.cls.cls #OA\_versus\_normal.cls.cls #OA\_versus\_normal\_repos |
| Phenotype | cls.cls#OA\_versus\_normal\_repos |
| Upregulated in class | OA |
| GeneSet | GOMF\_OXIDOREDUCTASE\_ACTIVITY\_ACTING\_ON\_THE\_CH\_CH\_GROUP\_OF\_DONORS\_NAD\_OR\_NADP\_AS\_ACCEPTOR |
| Enrichment Score (ES) | 0.36106598 |
| Normalized Enrichment Score (NES) | 1.237162 |
| Nominal p-value | 0.18181819 |
| FDR q-value | 0.47828352 |
| FWER p-Value | 1.0 |
Table: GSEA Results Summary

  

Fig 1: Enrichment plot: GOMF\_OXIDOREDUCTASE\_ACTIVITY\_ACTING\_ON\_THE\_CH\_CH\_GROUP\_OF\_DONORS\_NAD\_OR\_NADP\_AS\_ACCEPTOR      
 Profile of the Running ES Score & Positions of GeneSet Members on the Rank Ordered List

  

| SYMBOL | TITLE | RANK IN GENE LIST | RANK METRIC SCORE | RUNNING ES | CORE ENRICHMENT || 1 | BLVRA | biliverdin reductase A [Source:HGNC Symbol;Acc:HGNC:1062] | 942 | 0.529 | 0.0132 | Yes |
| 2 | TECR | "trans-2,3-enoyl-CoA reductase [Source:HGNC Symbol;Acc:HGNC:4551]" | 1047 | 0.508 | 0.0879 | Yes |
| 3 | BLVRB | biliverdin reductase B [Source:HGNC Symbol;Acc:HGNC:1063] | 1050 | 0.507 | 0.1704 | Yes |
| 4 | MECR | mitochondrial trans-2-enoyl-CoA reductase [Source:HGNC Symbol;Acc:HGNC:19691] | 1353 | 0.450 | 0.2204 | Yes |
| 5 | SRD5A1 | steroid 5 alpha-reductase 1 [Source:HGNC Symbol;Acc:HGNC:11284] | 1973 | 0.364 | 0.2316 | Yes |
| 6 | PECR | peroxisomal trans-2-enoyl-CoA reductase [Source:HGNC Symbol;Acc:HGNC:18281] | 1985 | 0.363 | 0.2899 | Yes |
| 7 | AKR1C3 | aldo-keto reductase family 1 member C3 [Source:HGNC Symbol;Acc:HGNC:386] | 1996 | 0.361 | 0.3480 | Yes |
| 8 | DHCR24 | 24-dehydrocholesterol reductase [Source:HGNC Symbol;Acc:HGNC:2859] | 3126 | 0.237 | 0.2990 | Yes |
| 9 | DECR1 | "2,4-dienoyl-CoA reductase 1 [Source:HGNC Symbol;Acc:HGNC:2753]" | 3272 | 0.224 | 0.3242 | Yes |
| 10 | ACADSB | acyl-CoA dehydrogenase short/branched chain [Source:HGNC Symbol;Acc:HGNC:91] | 3382 | 0.213 | 0.3506 | Yes |
| 11 | DPYD | dihydropyrimidine dehydrogenase [Source:HGNC Symbol;Acc:HGNC:3012] | 3830 | 0.173 | 0.3441 | Yes |
| 12 | BDH2 | 3-hydroxybutyrate dehydrogenase 2 [Source:HGNC Symbol;Acc:HGNC:32389] | 4247 | 0.133 | 0.3335 | Yes |
| 13 | TBXAS1 | thromboxane A synthase 1 [Source:HGNC Symbol;Acc:HGNC:11609] | 4338 | 0.125 | 0.3470 | Yes |
| 14 | TM7SF2 | transmembrane 7 superfamily member 2 [Source:HGNC Symbol;Acc:HGNC:11863] | 4410 | 0.120 | 0.3611 | Yes |
| 15 | AKR1D1 | aldo-keto reductase family 1 member D1 [Source:HGNC Symbol;Acc:HGNC:388] | 5836 | 0.006 | 0.2514 | No |
| 16 | PTGES2 | prostaglandin E synthase 2 [Source:HGNC Symbol;Acc:HGNC:17822] | 6269 | -0.025 | 0.2220 | No |
| 17 | SRD5A3 | steroid 5 alpha-reductase 3 [Source:HGNC Symbol;Acc:HGNC:25812] | 7325 | -0.104 | 0.1570 | No |
| 18 | DECR2 | "2,4-dienoyl-CoA reductase 2 [Source:HGNC Symbol;Acc:HGNC:2754]" | 7476 | -0.116 | 0.1644 | No |
| 19 | AKR1C1 | aldo-keto reductase family 1 member C1 [Source:HGNC Symbol;Acc:HGNC:384] | 8198 | -0.171 | 0.1363 | No |
| 20 | DHCR7 | 7-dehydrocholesterol reductase [Source:HGNC Symbol;Acc:HGNC:2860] | 8626 | -0.203 | 0.1361 | No |
| 21 | LBR | lamin B receptor [Source:HGNC Symbol;Acc:HGNC:6518] | 8834 | -0.220 | 0.1560 | No |
| 22 | SRD5A2 | steroid 5 alpha-reductase 2 [Source:HGNC Symbol;Acc:HGNC:11285] | 10149 | -0.336 | 0.1087 | No |
| 23 | FASN | fatty acid synthase [Source:HGNC Symbol;Acc:HGNC:3594] | 12194 | -0.643 | 0.0548 | No |
Table: GSEA details [plain text format]

  

Fig 2: GOMF\_OXIDOREDUCTASE\_ACTIVITY\_ACTING\_ON\_THE\_CH\_CH\_GROUP\_OF\_DONORS\_NAD\_OR\_NADP\_AS\_ACCEPTOR      
 Blue-Pink O' Gram in the Space of the Analyzed GeneSet

  

Fig 3: GOMF\_OXIDOREDUCTASE\_ACTIVITY\_ACTING\_ON\_THE\_CH\_CH\_GROUP\_OF\_DONORS\_NAD\_OR\_NADP\_AS\_ACCEPTOR: Random ES distribution      
 Gene set null distribution of ES for **GOMF\_OXIDOREDUCTASE\_ACTIVITY\_ACTING\_ON\_THE\_CH\_CH\_GROUP\_OF\_DONORS\_NAD\_OR\_NADP\_AS\_ACCEPTOR**
